# Real-Time Single Cell Monitoring: Measurement and Counting of Motile Sperm Using LCR Impedance-Integrated Microfluidic Device

**DOI:** 10.3390/mi10100647

**Published:** 2019-09-26

**Authors:** Chalinee Phiphattanaphiphop, Komgrit Leksakul, Rungrueang Phatthanakun, Apirak Suthummapiwat

**Affiliations:** 1 Ph.D’s Degree Program in Industrial Engineering, Department of Industrial Engineering, Faculty of Engineering, Chiang Mai University, Chiang Mai 50200, Thailand; rug_chalinee@hotmail.com; 2 Industrial Engineering Department, Chiang Mai University, Chiang Mai 50200, Thailand; 3 Synchrotron Light Research Institute (Public Organization), 111 University Avenue, Nakhon Ratchasima 30000, Thailand; Rungrueang@slri.or.th (R.P.); apirak@gmail.com (A.S.)

**Keywords:** real-time monitoring, sperm, motile sperm, LCR impedance, single cell counting, microfluidic system

## Abstract

In this research, we aimed to count the ratio of the number of motile to immotile sperm for patients with infertility problems based on a low-sperm-concentration examination. The microfluidic system consists of two series of applications: The conventional separation of motile sperm and the proposed inductance (L), capacitance (C), and resistance (R) or LCR impedance sperm counter. In the experiment, 96% of motile sperm were isolated from nonmotile sperm in the first part and transported to the second part to count and calculate real-time sperm concentration. A pair of microelectrodes composed of thin metal film were integrated between microchannels, resulting in a peak signal for LCR single-cell detection, as well as the estimated total sperm concentration. A minimum of 10 µL of the sperm sample was completely analyzed with an accuracy of 94.8% compared with the standard computer-assisted semen analysis (CASA) method. This method could be applied for low-cost sperm separation and counting in the future.

## 1. Introduction

Real-time sperm sorting and counting is ideal for men with fertility problems. Infertility is mainly related to semen quality, which, in most cases, is due to low sperm count, low sperm motility, and sperm abnormality. These factors reflect the ability of sperm to naturally fertilize an oocyte [1]. These deficiencies may be caused by stress, chronic inflammation, and environmental factors, such as exposure to particular toxins [2]. Semen is currently analyzed using laboratory tests that determine such values as the amount of semen secreted at the time of each ejaculation, number of sperm per milliliter, percentage of sperm with a normal shape, and infection of sperm. The normal values for each ejaculation are a volume more than 1.5 mL, more than 20 million sperm per milliliter, and more than 50 motile sperm with a normal shape. More than 30% of the patients have no infection, as evidenced by the lack of white blood cells in semen. The standard for concentration determination is the hemocytometer, but this labor-intensive method is only available in larger laboratories with an expensive computer-assisted semen analysis (CASA) system. Another method for estimating spermatozoa concentration is the use of flow cytometry [2,3]; antibody binding [4] and fluorescence labeling [5] are used to determine the concentration of progressive motile spermatozoa (>5 μm·s^−1^). The current treatment for patients faced with fertility problems is intracytoplasmic sperm injection (ICSI). Traditional in vitro fertilization (IVF) is playing an increasingly important role, but IVF is highly subjective and dependent on the skill of the embryologist [3,4]. ICSI is extensively used for sperm selection based on the swim-up and density gradient of sperm to select motile sperm from low-quality semen, whereas IVF is generally based on density-gradient centrifugation to select sperm cells. However, centrifugation in IVF has a negative effect on sperm viability and may result in DNA fragmentation [5]. Furthermore, these traditional techniques cannot be used for samples with low sperm counts (<4 million/mL, classified as oligospermia) [6], samples with low sperm motility oligospermia) [4], or cryopreserved sperm samples with reduced motility [7]. To estimate the success rate, it is crucial to consider human error in the process.

Microfabrication technologies have played an important role in the microfluidic system, which has led to developments in bio-sensing devices, including the sorting, manipulation, detection, and characterization of cells [8,9,10,11,12,13]. Ordinary detection methods in micro-devices include electrical and optical techniques, which generally require bulky and expensive instrumentation [14,15,16]. To apply the microfluidic chip in cooperation with assisted reproductive technology (ART), the electrical impedance spectroscope [17,18] was developed by integrating a microfluidic system with an electrical analyzer. This combination allows the chip to characterize cells for biomolecules [19,20,21] and cell analyses. This technique is applied extensively as a noninvasive and label-free detection of single cells [22,23]. Point-of-care testing is another important approach that was proposed for the design of faster microfluidic chips with higher sensitivity, and easy microchannel construction by means of polydimethylsiloxane (PDMS) was reported for biomedical applications [24,25]. The impedance detector in microfluidic devices was designed with the use of integrated microelectrodes to sense the impedance variation caused by the different dielectric properties within a small detection volume [26]. Microelectrodes offer numerous advantages compared with conventional large-area electrodes, such as high sensitivity, higher spatial resolution, and significant integration capability. Spermatozoa require the ability to analyze single-sperm motility and spatially confined single sperm cells. In most cases, sperm monitoring is based on optical analysis and a combination of integrated microelectrodes for electrical analysis. This system may provide a powerful tool for advanced sperm analysis and selection in ICSI applications [27,28].

In this study, we developed a simple microfluidic system for examining and sorting motile sperm and counting single sperm cells using microtechnology, laminar flow, and impedance variation detection using a measuring instrument (liquidity coverage ratio (LCR) meter). The isolation of motile from immotile sperm is based on laminar flow; the motile sperm have the ability to cross the streamline in a laminar fluid stream without external power or controls [29]. Then, the sperm are counted using impedance variation detection, measured with the inductance (L), capacitance (C), and resistance (R) or LCR method with integrated microelectrodes in the microfluidic system, known as the microfluidic chip. The impedance variation is caused by changes in the dielectric properties between the microelectrodes within a small detection volume. We integrated the results in a laboratory view program, which displays the peak impedance graph when the object passes through this real-time detector. Therefore, the chip can be used as a cell counter and to classify sample groups by using the number of impedance peaks and different impedance values, respectively. Signals are produced during the process of cells passing through the microelectrodes in the chip. This device not only counts the amount of sperm cells per unit time, but also counts in real time; the most current method only uses concentration of sperm for analysis [30]. This device can provide an accurate sperm count and can be applied to count other living cells.

## 2. Materials and Methods

### 2.1. Design and Fabrication

**Figure 1:**
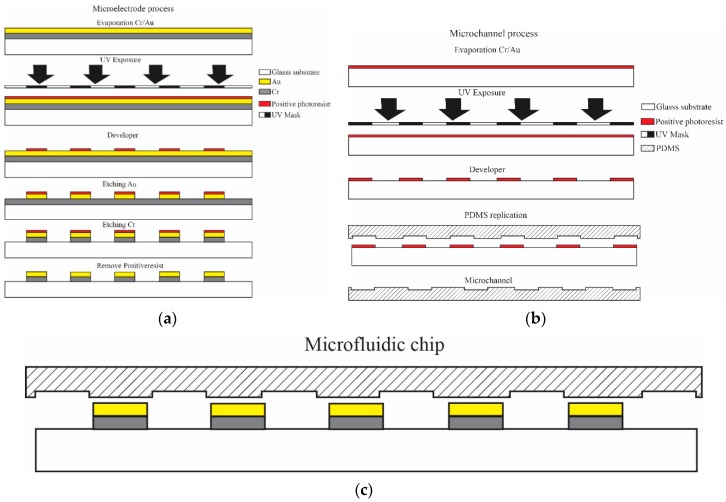

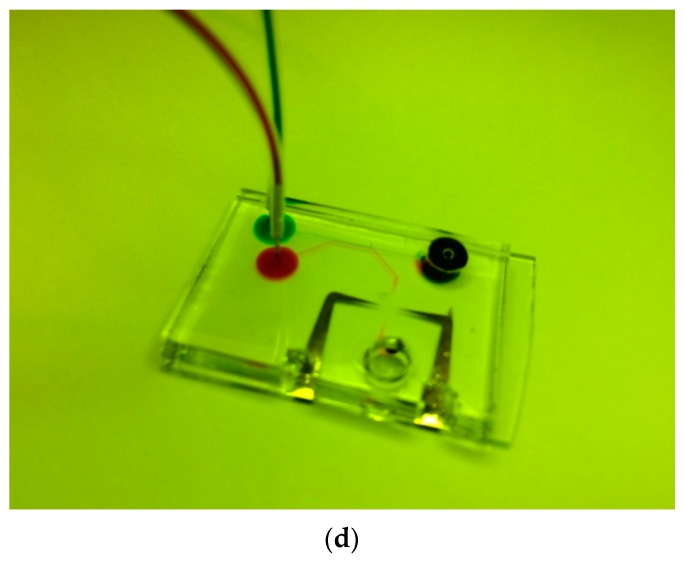
The fabrication of the microfluidic system for sorting and counting motile sperm. (**a**) Fabrication process of a pair of microelectrodes, (**b**) fabrication process of a polydimethylsiloxane (PDMS) microchannel, (**c**) assembly of microfluidic chip, and (**d**) a completed microfluidic system for real-time single cell monitoring.

The microfluidic system included a sorting system and counting system, created by integrating a single microchannel with a pair of microelectrodes. The system was patterned on a substrate, as illustrated in Figure 1. The microchannel was constructed from PDMS, which is compatible with the washing media and the sample solution, and easily replicated using soft lithography. The microchannel thickness was 50 µm with a width of 30 µm, allowing the cells to be arranged in a single line during the experiment. The microelectrodes were composed of Cr/Au with a 20 µm wide area on a substrate. The spatial distance between the microelectrodes was 30 µm. Based on this design, the microfluidic system consisted of a detection area in the space between the pair of microelectrodes. The LCR meter was connected to the electrodes to measure the cell impedances and simultaneously analyze the detected signal by using specific computer software. 

For the fabrication of the microfluidic system, two processes were conducted separately. First, a pair of Cr/Au microelectrodes were fabricated using UV lithography and wet etching in a clean room. Layers of 50 nm Cr and 50 nm Au were deposited onto a glass substrate via an evaporation method. The UV lithography process was applied to pattern the microelectrode areas. Wet chemical etching was used to produce the final form of the microelectrodes, as illustrated in Figure 1a. The electrode edges overlapped by 100 µm, as it was designed to support microchannel alignment and bonding. Thereafter, the PDMS microchannel was fabricated, as illustrated in Figure 1b. An SU-8 photoresist mold was created on the glass substrate using a standard UV lithography process. Following the development process, the microchannel mold, which was hard-baked at 100 °C, was used for the permanent pattern in the PDMS replication process. Finally, the replicated PDMS was punched at both microchannel ends and bonded to the microelectrodes using an oxygen plasma technique, as shown in Figure 1c. For the electrical circuit, the microfluidic chip was mounted onto a custom-made printed circuit board. Wire bonding was then applied to connect the microfluidic chip electrodes to the measurement systems (Figure 1d). The process was conducted under a microscope. 

### 2.2. Sample

In the experiment, an electrolyte semen extender medium [2] with a specific electrical conductivity of 2.3 Sm^−1^ was used. The extender medium was prepared with 8 g of NaCl, 0.2 g of KCl, 1.44 g of Na_2_HPO_4_, and 0.24 g (2 mM) of KH_2_PO_4_, which was dissolved in 800 mL of pure deionized (DI) water. After mixing well, the pH was adjusted to 7.4 with hydrochloric acid or sodium hydroxide using a pH meter (OAKTON Instruments, Vernon Hills, IL, USA). These media are generally maintained for the motility of bull sperm, following the necessary preprocessing steps. The bull samples were obtained from one straw, frozen, stored in liquid nitrogen (LN) vapor, and then stored in LN. The composition of a semen extender is mainly based on an energy resource from sugars, such as glucose and lactose, and a buffer medium of different inorganic or organic salts. For example, milk and egg yolk are basic ingredients in most extending media [6]. Egg yolk, in particular, is recognized as a protectant against cold shock, owing to its lipoprotein and phosphatidylcholine [8]. Semen extender solutions of approximately 20% egg yolk are standard for use in the majority of extenders. In the case of deep-frozen semen, glycerol is added for cryopreservation, and it remains the standard preservative agent. When semen is frozen in a glass ampoule via conventional methods, approximately 7% glycerol is optimal for the egg yolk citrate (EYC) and Tris extender, and 11% to 13% for fresh and reconstituted skim milk [31]. The addition of glycerol may enhance motility, although the advantage gained is generally limited [32]. The average sperm production of a bull varies between 3.6 × 10^9^ and 12 × 10^9^ sperm per ejaculate. When using liquid semen, a minimum amount of 2.5 million sperm per dose is required; consequently, each ejaculation can provide 1440 to 4800 doses. However, when using frozen semen, approximately 20 million total sperm per dose are required, which reduces the sample amount to 180–600 doses of frozen semen per ejaculation. The medium was warmed in the water batch at 37 °C for 30 min. Then, the frozen straw was removed from the LN and maintained in the medium for 40 s. Thereafter, the medium was stored in a constant-temperature chamber at 37 °C. Dilute bull sperm with a medium in the ratio of 1 µL:100 µL was used in this experiment.

### 2.3. Impedance Detection and Analysis

#### 2.3.1. Overview of LCR Impedance Program

The impedance detection and analysis were designed using the LabVIEW program (National Instruments, Austin, Taxas, USA), which is convenient for editing and designing graphical user interfaces (GUIs). The main objective was to measure the cell impedance and count the semen related to the LCR value, which can be calculated as:

(1) $$\begin{array}{c} Z=R+jx\\ x=\frac{1}{\omega C}+\omega L\;,\;\omega =2\pi f\\ so\;that,Z=\sqrt{R^{2}+x^{2}} \end{array}$$

where *Z* is the complex impedance, *R* is the real part of the impedance (Ω), *jx* is the imaginary part, *x* is the reactance value, ω is angular frequency, f is the frequency, *L* is inductance, and *C* is capacitance.

**Figure 2:**
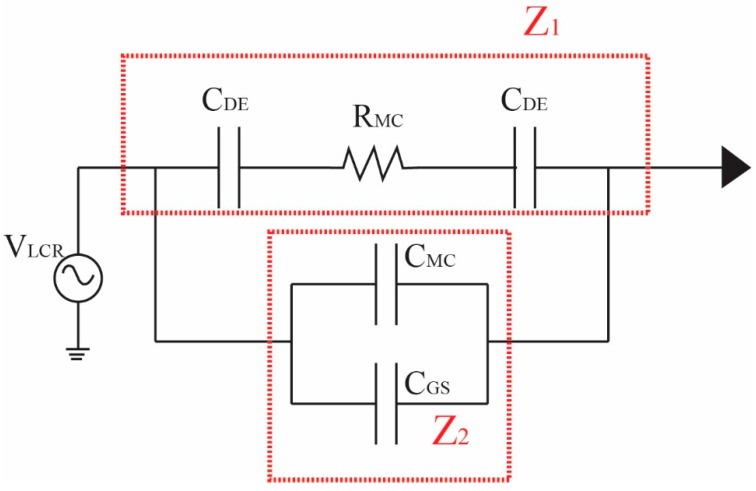
The electrical equivalent circuit of a microfluidic chip.

**Figure 3:**
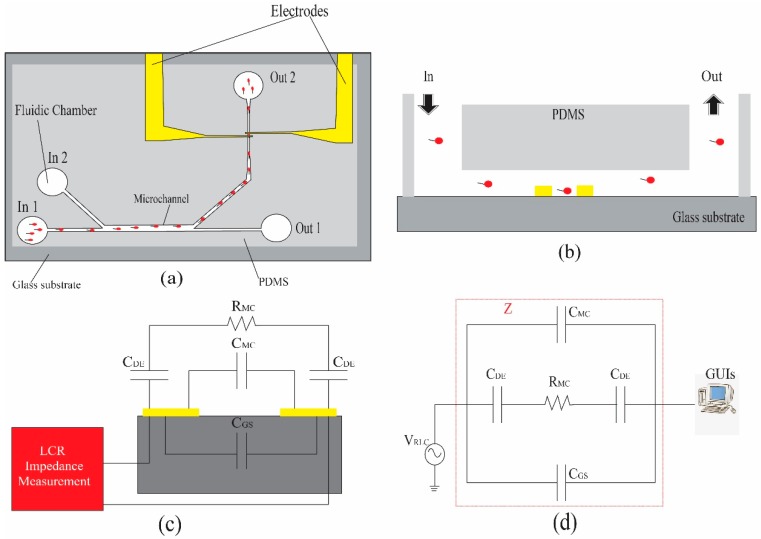
A schematic of the microfluidic chip sorting, counting, and detecting sperm: (**a**) top view and (**b**) sectioned front view. (**c**) Electrical equivalent circuit of the microfluidic sperm counting device. (**d**) The measurement setting was used for sperm and particle detection.

Based on the impedance method, when cells pass through the microelectrodes, increasing impedance will generate peak signals that are higher than the initial value. The signals were read with an LCR meter, as used in particle testing in microfluidic chips. This can be explained by the theory of resistance, as follows:

(2) $$\frac{1}{Z_{t}}=\frac{1}{Z_{1}}+\frac{1}{Z_{2}}$$

where *Z_t_* is the magnitude of the total impedance within the system at freguency $f_{m}$, which can be written as a parallel combination of the magnitudes of two impedances, *Z*_1_ and *Z*_2_. *Z*_1_ is the magnitude of the impedance between the dielectrode capacitances, *C*_DE_, which are formed at the interfaces of the electrolyte with the electrode and in series with the microchannel resistance *R*_MC_. *Z*_2_ is the magnitude of the impedance between the microchannel capacitance *C*_MC_ through the electrolyte and the stray capacitance through the gas substrate, which is *C*_GS_, as shown by the electrical equivalent circuit of the microfluidic chip in Figure 2. The impedance equation can be described in Equations (3) and (4). A schematic of the microfluidic chip applied with ac voltage to a pair of electrodes, V_LCR_, for counting and detecting sperm and particles is shown in Figure 3.

(3) $$\begin{array}{ll} Z_{1} & =R_{MC}+(\frac{1}{j\omega C_{DE}}+\frac{1}{j\omega C_{DE}})\\ & =R_{MC}+(\frac{2}{j\omega C_{DE}})\;\;;\omega =2\pi f\\ & =\;R_{MC}+\frac{2}{j2\pi fC_{DE}}\\ & =\;R_{MC}+\frac{1}{j\pi fC_{DE}} \end{array}$$

(4) $$\begin{array}{ll} Z_{2} & =\;\frac{1}{j\omega \left(C_{MC}+C_{GS}\right)}\;,\;C_{EF}=C_{MC}+C_{GS}\\ & =\;\frac{1}{j\omega C_{EF}}\\ & =\;\frac{1}{j2\pi fC_{EF}} \end{array}$$

In Figure 3a, the motile sperm are first isolated by feeding the sperm sample into reservior In 1, which includes motile sperm, immotile sperm, and celluar debris. The first laminar flow stream conveys them to resevior Out 1. The second laminar stream is generated from the media sample, which originates from resevoir In 2 and allows the motile sperm to swim across the streamline from the first laminar stream to resevior Out 2, while immotile sperm remain in the first larminar flow stream. The flow rate of In 1, which was the sperm sample, was 0.24 µL/min, whereas the medium sample, or In 2, used a flow rate of 0.34 µL/min. The parameters of the microfluidic chip were as follows: (1) The angle between the microchannels was 43°, (2) the width of the inlet and outlet microchannel was 200 µm, (3) the width of the separation microchannel was 400 µm, and (4) the thickness of the microchannel was 50 µm. After the motile sperm were isolated from the immotile sperm in the laminar fluid stream, they flowed through the pair of electrodes, as shown in Figure 3b. The speed of real-time signal processing in impedance measurement was between 20 Hz and 2 MHz or 20 ms to 0.5 µs, which covered all the motile sperm movement while the sperm flowed through the pair of electrodes. The impedance measurement, therefore, depended on the characteristics of the sperm or particle between the pair of electrodes. The two-stream flows in the microchannel did not mix together, which allowed motile sperm to swim across the main streamline to another, while immotile sperm and cellular debris remained in the main streamline. The sorting mechanism of the motile sperm can be simply applied based on the ability of motile sperm to cross streamlines in a laminar fluid stream, which was different from Segerink et al. [13], who applied it for nonmoving cells and sorting by using a complicated microstructure and techniques such as fluorescent label-based, bead-based, and label-free sorting. This microfluidic device can be used for cell separation of motile sperm in the future, as previously suggested [30].

#### 2.3.2. Detection Method for LCR Impedance and Counting Spermatozoa

The signals from the experiment were analyzed by the LCR impedance program, obtained from the device connected to the LCR meter. The sample was injected into the inlet and passed through the microchannel and the pair of electrodes. For peak impedance, when the particles pass through the electrodes, the signal increases to higher than the normal amplitude. The analysis program can set parameters and conditions to count particles or spermatozoa. All results and data can be displayed in real time on the display panel in the program. The raw data are automatically saved, using specific path names defined by the user. 

#### 2.3.3. Programming and GUI 

LabVIEW software was used to design the GUI with three input sections: Parameter setting, control mode, and real-time display. Details are provided in the Supplementary Materials.

## 3. Results and Discussion

**Figure 4:**
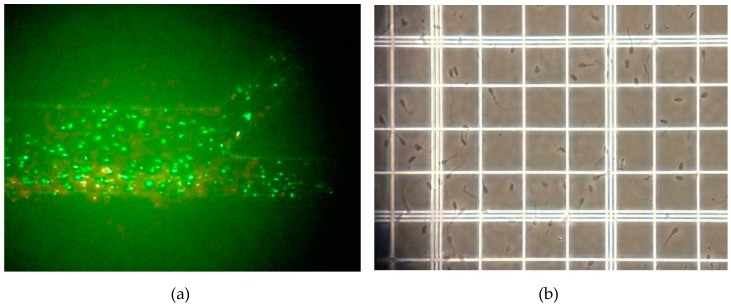
Isolation of motile sperm from immotile sperm and cellular debris in two laminar fluid streams. (**a**) Red and green represent immotile and motile sperm, repectively; and (**b**) determining the concentration of the motile sperm using a hemocytometer.

Semen and media were loaded into the inlet reservoirs of the microfluidic chip using a BS-8000 syringe pump (Braintree Scientific, Inc., Braintree, MA, USA) after the semen was incubated at room temperature for 30 min to reduce its viscosity. The sperm sample was then flown along the first laminar flow stream, and the motile sperm gradullay swam across the streamline to the second flow stream and were conveyed to the angle microchannel, as shown in Figure 4a. The concentration of the motile sperm at the outlet resevoir was determined using a hemocytometer, as shown in Figure 4b. The average number of motile sperm at Out 1 and Out 2 were 7.1 × 10^5^ cells and 3.1 × 10^4^ cells, respectively. Therefore, 96% of motile sperm were successfully isolated in the first part and transported to the second part for counting and calculating the real-time sperm concentration. 

**Figure 5:**
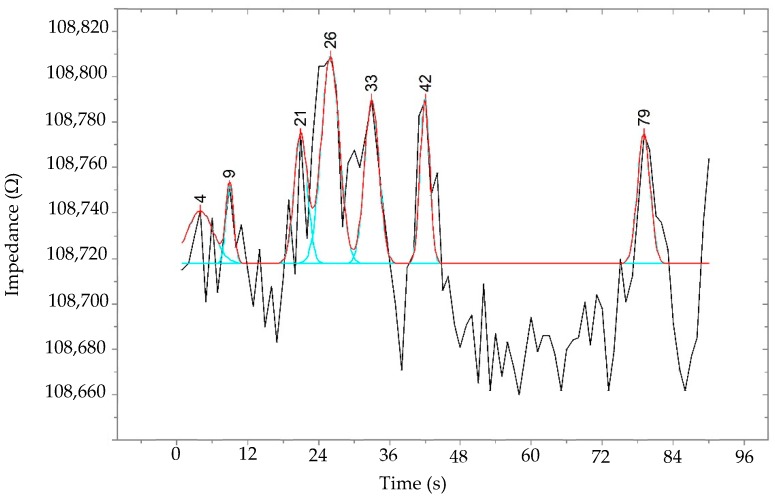
The peak impedance distribution of sperm when passing the electrode, showing the number of times which the motile sperm signals were detected within 90 s.

**Figure 6:**
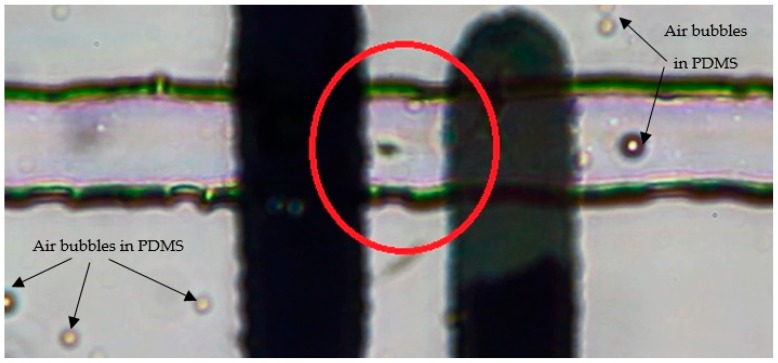
The sperm cell passing between the two electrodes.

**Figure 7:**
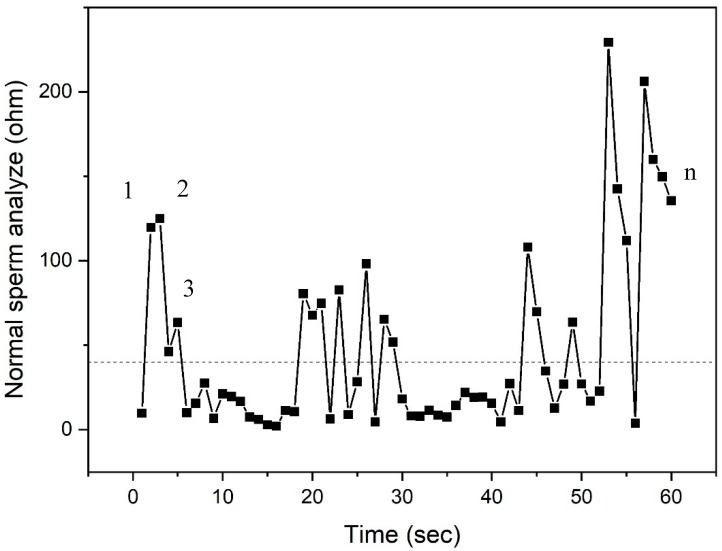
The results of the measurement of complex resistance when sperm flows through the sperm count device in real time. The program can display the results according to the actual number and/or as a number of strong sperm per time (1, 2, 3 … *n* in seconds).

**Figure 8:**
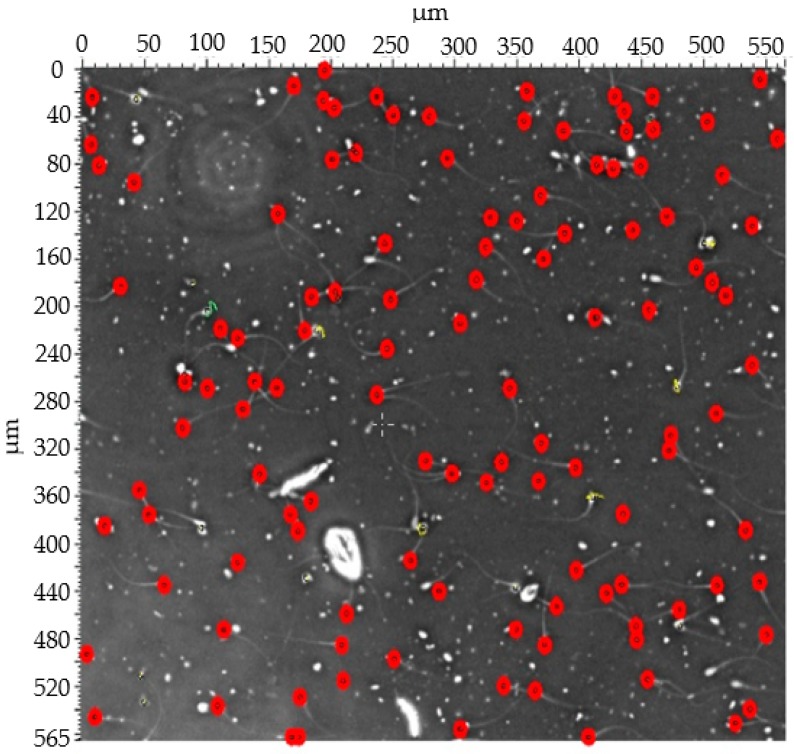
The sample was investigated via computer-assisted sperm analysis (CASA), which is standard for semen analysis.

The spermatozoa sort motile sperm, which swim up through the electrodes, causing a change in the electrical impedance signal, as observed from the results of the synchronization of the video comparing to images of measurement data. The motile sperm have an average path velocity (VAP) of >5 μm/s. The experimental results show that the average electrical impedance of sperm for operating processes was 110.54 kΩ (*p* < 0.05). The standard deviation (SD) of sperm was 153.7, the coefficient of variation (CV) was 1.39%, and the adjusted coefficient of determination (*R*^2^) was greater than 99.97%, which is a high score for reliability, demonstrating that this information is reliable. The statistical analysis was performed using Minitab16 (Minitab Pty Ltd., Sydney, Australia), as shown in Table 1. These data are normally distributed. The peak impedance was analyzed using originPro2016^@^ (OriginLab Corporation, Northampton, MA, USA), and the peak impedance distribution is shown in Figure 5. The sperm was detected and counted under microscopy, as shown in Figure 6. The variation of the device was 99.99 when used in the measurement of impedance with four replications, with an error less than 0.88%, as shown in Table 2. This method can be used as a cell counter when the sperm flow passes the area of the detected electrodes, as illustrated in Figure 7. Subsequently, the sample was investigated via computer-assisted sperm analysis (CASA), which is standard for semen analysis [26], as illustrated in Figure 8. 

The efficiency of the device in detecting and counting human sperm was validated. 

Table 3 provides a comparison of sperm counting via CASA analysis and LCR impedance. Under the same experimental conditions, the CASA system, which is based on image analysis, examines both living and nonliving sperm and reports an average motile sperm count of 517 with an operation time between 15 and 20 min. LCR impedance integrated into microfluidic devices was designed with the objective of motile sperm isolation and counting, which can be performed within 10 min, resulting in an average motile sperm count of 488. The validation of LCR impedance compared with the CASA system was 94.8%, which was slightly different from the CASA system, since some of the motile sperm in the microfluidic chip leaked and mixed with immotile sperm. However, the proposed LCR impedance integrated with a microfluidic chip was compact and simple, so it could be applied in a portable device in the future. 

## 4. Conclusions

In this study, an effective device for motile sperm sorting and counting using an LCR impedance-integrated microfluidic was devised. The motile sperm are sorted via laminar flow, and counting is achieved by using peak impedance; when the particles pass through the electrodes, the signal increases above the normal amplitude difference. The program displays the results according to the actual count or as the number of strong sperm per unit time. The experimental results showed that the average electrical impedance of sperm for the operating process is 110.54 kΩ (*p* < 0.05). The SD of sperm was 153.7, the CV was 1.39%, and the adjusted *R*^2^ was greater than 99.97%, which is a high reliability score, supporting the reliability of the information. The results demonstrated that the different impedance signals of micron-sized objects passing through the microelectrodes can be counted as a number of cells. This device can measure approximately 488 cells in a in 10 µL sperm sample in comparison with the CASA standard, with the accuracy of 94.8%. 

## Figures and Tables

**Table 1:** One-way ANOVA analysis using Minitab16.

Source	DF	SS	MS	*F*	*p*
**Factor**	1	4,435,877,635	24,435,877,635	20,697.51	0.000
**Error**	6	7,083,715	1,180,619	-	-
**Total**	7	24,442,961,351	-	-	-
***S* = 1087***R*^2^ = **99.97%** *R*^2^ **(adj) = 99.97%**					

DF is degrees of freedom; SS is sum of squares; MS is mean squares; *F* is *F* test, which was used to test if a relationship exists between the dependent and independent variable, a statistic based on the *F* distribution is used; *S* is summary of model; and *P* is *p*-value, which is the probability that an equal amount of variation in the dependent variable would be observed in the case that the independent variable does not affect the dependent variable.

**Table 2:** Experimental results showing the average impedance of sperm.

Sample	Baseline Impedance (kΩ)	Standard Error	Count/90 s
1	112.48	1.76%	37
2	110.49	0.05%	42
3	110.47	0.06%	32
4	108.72	1.65%	40
Average	110.54	0.88%	38

**Table 3:** Comparison between CASA and liquidity coverage ratio (LCR) impedance for spermatozoa detection and counting.

Analysis	Volume (µL)	Time (min)	Total No. of Sperm Analyzed	Method	Validation (100%)
CASA	10	15–20	517	Image Programming	Reference Standard 100%
LCR Impedance	10	5–10	488	Difference in Impedance	94.8%

## Supplementary Materials

The following are available online at https://www.mdpi.com/2072-666X/10/10/647/s1, Figure S1: GUI of RLC measurement program.
