# Supplementary material for: Real-Time Single Cell Monitoring: Measurement and Counting of Motile Sperm Using LCR Impedance-Integrated Microfluidic Device
Source: Micromachines (Basel). 2019 Sep 26;10(10):647. doi: 10.3390/mi10100647 (PMC6843681; doi:10.3390/mi10100647)
Supplement: Supplementary file 1 [file micromachines-10-00647-s001.pdf]

Supplementary Materials

Real-Time Single Cell Monitoring: Measurement and Counting of Motile Sperm Using LCR Impedance-Integrated Microfluidic Device

Chalinee Phiphattanaphiphop [1,2], Komgrit Leksakul [2,*], Rungrueang Phatthanakun [3] and Apirak Suthummapiwat [3]

1. Ph.D's Degree Program in Industrial Engineering, Department of Industrial Engineering, Faculty of Engineering, Chiang Mai University, Chiang Mai 50200, Thailand
2. Industrial Engineering Department, Chiang Mai University, Chiang Mai 50200, Thailand
3. Synchrotron Light Research Institute (Public Organization), 111 University Avenue, Nakhon Ratchasima 30000, Thailand

* Correspondence: komgrit@eng.cmu.ac.th

Programming and GUI for Counting Sperm

The first section was designed for the parameter settings. The resource name requires the user to define the machine model, indicating the Key sight (formerly Agilent, Santa Clara, CA, USA) E4980A Precision LCR Meter (20 Hz to 2 MHz). The machine can select 23 data points in total, based on user-investigated parameters, such as the Z-thd for measuring the impedance, and *Cp* and *Rp* for measuring the capacitance and resistance, respectively. The range display can be set automatically in the case of long investigation times of both voltage and current signal types.

The second section was implemented to define the time interval, raw data, and graphic recordings, resulting in the number of particles passing through the detection area of the microelectrodes.

The final section is a real-time experimental display related to the user-defined functions in the first section, including Z-thd as well as data normalization, which is described as

$$y = x_n - x_{n-1} \tag{S1}$$

where y is a variable parameter, x_n is the amplitude at the present time, and x_{n-1} is the previous amplitude at time n-1.

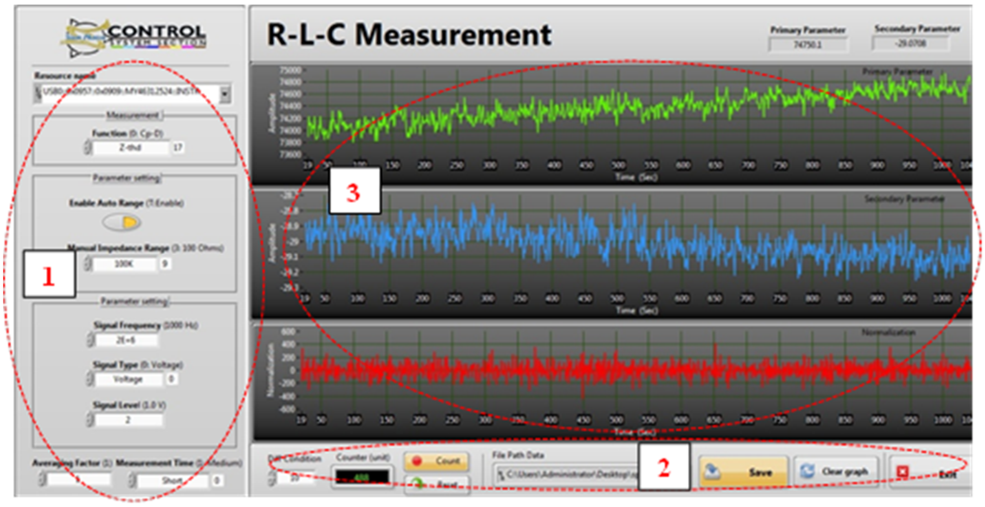

Figure S1 GUI of RLC measurement program.
